# Prevalence and factors associated with adolescent pregnancy in Hoima district, Uganda: A community-based cross-sectional study

**DOI:** 10.1371/journal.pgph.0005331

**Published:** 2026-07-15

**Authors:** Joyce Wamakote Wandeka, Ronald Kooko, Justine Bukenya, Rogers Kisame

**Affiliations:** 1 Department of Community Health and Behavioral Science, School of public health, Makerere University, Kampala, Uganda; 2 Department of Integrated Epidemiology, Ministry of Health-Uganda, Surveillance and Public Health Emergencies, Kampala, Uganda; 3 Department of Global Health Security, Baylor College of Medicine Children’s Foundation Uganda, Kampala, Uganda; International Rescue Committee, KENYA

## Abstract

Adolescent pregnancy is a global public health problem with serious social and medical implications relating to maternal and child health. In Uganda, particularly Hoima district, limited information about the factors influencing adolescent pregnancy is available. The study estimated the prevalence of adolescent pregnancy and identified factors independently associated with adolescent pregnancy among girls aged 13–19 years in Hoima district. We conducted a community-based cross-sectional study among 543 adolescent girls randomly selected using multistage sampling. Data were collected using a structured, interviewer-administered questionnaire. Bivariate logistic regression was used to assess associations between independent variables and adolescent pregnancy. Variables with p < 0.20 at bivariate analysis were included in a multivariable logistic regression model to identify factors independently associated with adolescent pregnancy. Adjusted odds ratios (AORs) with 95% confidence intervals (CIs) were reported, and statistical significance was set at p < 0.05. Findings revealed 29.1% prevalence of adolescent pregnancy. Age (18–19 years) AOR 4.4 (95%CI 1.5–12.8), low parents’ economic status AOR 5.4 (95%CI 2.5–11.8), multiple sexual partners AOR 8.0 (95%CI 4.5–14.2), being out of school AOR 12.0 (95%CI 5.0–29.1), early marriage AOR 37.0 (95%CI 13.4–107.5), having no control over sex AOR 7.4 (95%CI 3.7-16.4), not discussing SRH with parents AOR 8.4 (95%CI 3.3–21.5), witnessed domestic violence AOR 30.0 (95%CI 12.0–77.5), never received counselling AOR 5.7 (95%CI 3.6–9.0), and, rural residence AOR 1.8 (95%CI 1.2–2.9) were significant predictors of adolescent pregnancy. These findings suggest that adolescent pregnancy in Hoima district remains high and is associated with interconnected individual, family, health system, and community-level factors. Interventions should prioritize keeping girls in school, strengthening adolescent-friendly and non-judgmental health services, addressing household poverty, preventing early marriage, and promoting supportive family and community environments to reduce adolescent pregnancy in rural Ugandan settings.

## Introduction

Adolescent pregnancy, defined by the United Nations International Children’s Emergency Fund (UNICEF) as pregnancy occurring among girls aged 10–19 years, remains a major public health and social concern globally, particularly in low- and middle-income countries (LMICs) [1,2]. An estimated 21 million girls aged 15–19 years become pregnant annually in LMICs, nearly half of which are unintended, resulting in approximately 12 million births each year [3]. These pregnancies are associated with adverse maternal, neonatal, and socio-economic outcomes that undermine girls’ health, education, and long-term wellbeing.

Sub-Saharan Africa bears the highest burden of adolescent pregnancy worldwide, with adolescent birth rates remaining substantially higher than global averages despite recent declines elsewhere [4]. Uganda in general and Hoima district in particular continue to report one of the highest adolescent pregnancy rates, estimated at approximately 24.0% and 30.6% respectively, posing a significant challenge to local health systems, communities, and development efforts [5,6].

Despite the implementation of policies and related laws such as the Uganda’s National Policy Guidelines and Service Standards for Sexual and Reproductive Health and Rights (2017) emphasizing reduction of adolescent pregnancies by increasing access to reproductive health services, the Penal Code Act criminalizing sexual exploitation of minors, and the school health policy that advocates for comprehensive sex education to mitigate early pregnancies, adolescent pregnancies remain relatively high in Uganda, especially in Hoima district [6,7].

Therefore, to address the impact of adolescent pregnancies, there is a need to continuously understand the risk factors associated with adolescent pregnancy. Despite the high prevalence of adolescent pregnancies in Hoima district, previous research on the sexual and reproductive health was limited to in-school adolescent girls’ population with less focus on community-based studies thus neglecting adolescents who are out of school for various socio-economic or personal reasons [6].

Even though numerous studies have been done in Uganda to identify a wide variety of risk factors associated with adolescent pregnancies, there is a need for context-specific evidence to inform locally feasible interventions to reduce adolescent pregnancies in Hoima district. This study therefore assessed the prevalence of adolescent pregnancies and factors associated among adolescent in Hoima district, Uganda. The findings from this study will support policymakers and stakeholders to strengthen adolescent reproductive health interventions and inform the implementation of existing policies such as the National Health Policy, School Health Policy, and Adolescent Health Policy.

## Materials and methods

### Ethical considerations

This study was reviewed and approved by the Makerere University School of Public Health Research and Ethics Committee under reference number; MakSPH-REC_330. Permission to conduct the study was also obtained from Hoima District Health Officer (DHO) and the District Health Team (DHT). Before recruitment, the purpose and procedures of the study were explained to potential participants, and participation was voluntary. Written informed consent was obtained from all adult participants (≥18 years) and for participants younger than 18 years, written informed consent was obtained from their parents or legal guardians, and assent was obtained from the minors themselves prior to completing the questionnaire. Confidentiality was maintained throughout the study, and all information collected was used solely for research purposes.

### Study design and setting

This was a community-based cross-sectional study involving quantitative data collection approaches in three sub-counties of Hoima district: Buseruka, Buhanika, and Kitoba. The district is located in the mid-western region of Uganda and it is predominantly rural, with livelihoods mainly based on subsistence farming, fishing, and small-scale trade. The district has documented high levels of adolescent pregnancy compared to the national average, attributed to early marriage practices, school dropout, poverty, and limited access to adolescent-friendly sexual and reproductive health (SRH) services.

### Study population

Adolescent girls aged 13–19 years who had resided in the selected areas for at least 12 months prior to the study to ensure participants had adequate exposure to local contextual factors influencing adolescent pregnancy and to reduce bias from recent migrants.

### Inclusion criteria

The study included girls aged 13–19 years who had resided in the selected households for at least 12 months, provided assent, and those younger than 18 years whose parent or guardian provided consent.

### Exclusion criteria

The study excluded girls who were severely ill or cognitively unable to respond to the questionnaire.

### Sample size determination

The sample size formula for Kish Leslie (1965) was used as expressed below,


$$\begin{array}{c} \text{Sample size},\text{\textasciitilde{}}n\;=\frac{Z^{\text{2}}\times P(\text{1}-P)}{d^{\text{2}}} \end{array}$$

Where Z is the standard normal deviate of 1.96 (95% confidence interval)

P= Prevalence of adolescent pregnancies in Hoima district at 30.6% [6].

d = level of precision (+/- 5%)


$$\begin{array}{c} n\;=\frac{{\text{1}.\text{9}\text{6}}^{\text{2}}\times \text{0}.\text{3}\text{0}\text{6}\;(\text{1}-\text{0}.\text{3}\text{0}\text{6})}{{\text{0}.\text{0}\text{5}}^{\text{2}}}\;=\text{326 adolescents} \end{array}$$

Considering a non-response rate of 10%

Final sample size= Effective sample size/ (1- non response rate anticipated)

n= 326/ (1-10%) ~ 362 adolescents

To account for cluster sampling, a design effect of 1.5 was applied, consistent with community-based surveys where intra-cluster correlation is expected.

Therefore, considering a design effect of 1.5, n=543

### Sampling procedure

Multi-stage random sampling was used to select study respondents. Hoima district is divided into two counties, Bugahya and Kigorobya. The counties are sub divided into 5 sub counties and 1 Town Council namely; Bugahya County – Buseruka, Buhanika, Kitoba and Kyabagambire sub counties; Kigorobya County – Kigorobya Sub County and Kigorobya Town Council. Three sub counties (Buseruka, Buhanika, Kitoba) were randomly selected by simple random sampling using rotary method to participate in the study. The principal investigator then randomly selected half of the parishes from each selected sub-county. Here the names of all parishes in each selected sub-county were written on individual pieces of paper, folded, and placed in a container. Half of the parishes were then drawn from the container without replacement for participation in the study and this sampling technique was also applied at parish level to select half the number of villages in each parish to participate in the study. From each village, a list of households was obtained and systematic random sampling was used to select households. In households with more than one eligible adolescent girl, simple random selection was applied to select one respondent. The number of participants selected per village was determined using probability proportional to size (PPS) based on the estimated number of adolescent girls aged 13–19 years per village. Village population data were obtained from the Hoima district population officer (Table 1).

**Table 1:** Sampling criteria for study participants.

Village	Sampling frame (number of adolescent girls)	Proportion of adolescent girls aged 13–19	Sample size
Ikoba	109	0.0588	32
Kabango	97	0.0523	28
Kidubi	83	0.0448	24
Kitoole	72	0.0388	21
Kabaale	91	0.0491	27
Zorobi	76	0.0410	22
Bank cell	112	0.0604	34
Kibati	121	0.0653	36
Park cell	107	0.0577	31
Market cell	113	0.0609	34
Kihooko	98	0.0529	29
Kitinduru	87	0.0469	25
Kyabicwe	76	0.0410	22
Kikimizi	87	0.0469	25
Busigi	99	0.0534	29
Nsonga	83	0.0448	24
Kiina	72	0.0388	21
Kacunde	96	0.0518	28
Susa	81	0.0437	24
Kyakapere	94	0.0507	27
**Total**	**1854**	**1**	**543**

Data from Hoima district population office, 2024.

### Study variables

The primary outcome variable was adolescent pregnancy, operationally defined as self-reported current pregnancy or ever been pregnant. Pregnancy status was determined through self-report, as biological verification was not feasible in this community-based study.

Independent variables included: Socio-demographic factors (age, residence, education), Individual factors (school attendance, sexual partners, control over sex), Family factors (parent-child communication, domestic violence), Health facility factors (youth-friendly services, counselling), and Community factors (peer influence, residence).

### Data collection

Trained research assistants with support from the principal investigator collected data for a period of one month from 6^th^ March 2024 to 6^th^ April 2024 using a pre-tested interviewer administered structured questionnaire. Local leaders and village health teams only facilitated community entry and did not participate in data collection to minimize social desirability bias. The questionnaire was adapted from validated tools used in similar studies [6,7] and customized to meet the study objectives. The questionnaire covered socio-demographic characteristics, sexual behavior, family environment, health service access, and pregnancy history.

### Training of research assistants

The principal investigator selected the research assistants, trained them for four days on questionnaire administration, data collection, and ethical considerations; they were also trained in effective communication skills, showing empathy and never being judgmental. The research assistants were aged between 20 and 30 years and fluent in the local language and attained at least certificate qualification with some holding diploma and bachelor’s degree in health-related or social science field. This helped to ensure free, open communication for the participants and reliable administration of the questionnaire.

### Quality control measures

The questionnaire was translated into Runyoro and back-translated into English to ensure semantic consistency. Pretesting was conducted among 20 adolescent girls in Kakumiro District, which has similar socio-demographic characteristics to Hoima.

Internal consistency reliability was assessed using Cronbach’s alpha, yielding a coefficient of ≥0.78 across key domains, indicating acceptable reliability. Feedback from pretesting informed refinement of question wording and sequencing. Fieldwork was overseen by the principal investigator to ensure completeness and accuracy of data collected.

### Data analysis

Data analysis was performed using Statistical Package for Social Sciences (SPSS) version 26. Descriptive statistics were used to summarize participants’ characteristics, with categorical variables presented as frequencies and percentages. Prior to multivariable modeling, multicollinearity among independent variables was assessed using the variance inflation factor (VIF), and all variables had VIF values below 5, indicating absence of significant multicollinearity. Bivariate logistic regression analysis was conducted to examine the association between each independent variable and adolescent pregnancy. Variables with a p-value < 0.20 at the bivariate level were retained for inclusion in the multivariable logistic regression model in order to avoid excluding potential predictors. Multivariable logistic regression analysis was then performed to identify factors independently associated with adolescent pregnancy, and adjusted odds ratios (AORs) with corresponding 95% confidence intervals were reported. Statistical significance was set at p < 0.05. Model fit was assessed using the Hosmer–Lemeshow goodness-of-fit test (χ² = 1.20, p = 0.99), indicating adequate model fit.

## Results

### Sociodemographic characteristics of participants

In this study, data from 543 participants were analyzed. The mean age was 15.9 ± 1.9 years and the median age was 16 years, with an interquartile range of 14–17 years old.

The majority of participants (61.1%) included in this study were living in rural areas, over 44.0% were between the ages of 15–17 years and 24.1% between the ages of 18–19 years, majority of the respondents 58.8% had attained primary level of education, 33.0% of them were married and 39.0% had multiple sexual partners.

About two-thirds, (77.2%) of the parents whose adolescents were included the study had low/poor economic status, a high number (66.1%) of participants had both parents alive and catholic was the dominant religion at 34.4%. Approximately, (44.4) % of the adolescents had witnessed domestic violence. There was almost a uniform sample distribution among the sub-counties, Buhanika, Buseruka and Kitoba at 34.4%, 33.0% and 32.6% respectively (Table 2)

**Table 2:** Socio-demographic characteristics of the respondents (n = 543).

Variable	Frequency	Percentage
**Age (Years)**		
**Age (mean ± SD)**	15.9 ± 1.9	–
**Age (Median, IQR)**	16 (14, 17)	–
13-14	172	31.7
15-17	240	44.2
18-19	131	24.1
**Sub-county**		
Buhanika	187	34.4
Buseruka	179	33.0
Kitoba	177	32.6
**Place of residence**		
Rural	333	61.1
Urban	221	38.9
**Ethnicity**		
Muganda	76	14.0
Munyoro	301	55.4
Musoga	64	11.8
Mutooro	14	2.6
Others	88	16.2
**Early marriage**		
No	364	67.0
Yes	179	33.0
**Multiple sexual partners**		
No	331	61.0
Yes	212	39.0
**Level of Education (Girl)**		
No formal education	12	2.2
Primary education	319	58.8
Secondary education	212	39.0
**Religion**		
Anglican	132	24.3
Catholic	187	34.4
Muslim	67	12.3
Pentecostal	157	28.9
**Parents’ economic status**		
High	124	22.8
Low	419	77.2
**Parents alive**		
No	184	33.9
Yes	359	66.1
**Witnessed domestic violence**		
No	302	55.6
Yes	241	44.4

### Prevalence of adolescent pregnancies in Hoima district

Out of 543 adolescent girls included in the study, 158 reported having ever been pregnant, giving a prevalence of 29.1%.

### Factors associated with adolescent pregnancies in Hoima district (Unadjusted analysis)

#### Sociodemographic characteristics of respondents.

In this study, age was significantly associated with adolescent pregnancy. There was a significantly high prevalence of adolescent pregnancy among adolescents aged 18–19 at 64.9% compared to 4.7% among those aged 13–14. Adolescents aged 18–19 were 37 times more likely to get pregnant as compared to adolescents aged 13–14 COR 37.0 (95%CI 17.1-83.9). The prevalence of adolescent pregnancy among adolescents who were married was high at 74.1% and 25 times more likely to get pregnant than their counterparts who were not married COR 25.0 (95%CI 15.7-41.6). The education of the caretaker/parent was significantly associated with adolescent pregnancy with about 45.6% pregnancies happening among adolescents whose parents had no formal education and 18.1% among those whose parents had at least attained secondary level of education. There was a substantially high proportion of adolescent pregnancies 34.4% among adolescents whose parents fall under a low (poor) economic status strata compared to 11.3% whose parents belong to a high (rich) economics status. Adolescents whose parents were poor were 4.1 times more likely to report having ever been pregnant compared to those whose parents were rich COR 4.1 (95%CI 2.3-7.4). More than half of adolescent pregnancies 54.9% were among adolescents who did not have both biological parents alive and these adolescents were 6.4 times more like to get pregnant than their counterparts who had both parents alive COR 6.4 (95%CI 4.3-9.7) (Table 3).

**Table 3:** Unadjusted analysis of the socio-demographic characteristics associated with adolescent pregnancy.

Variable	Adolescent pregnancy		Crude OR (95% CI)	P-value
	No N = 385 (%)	Yes N = 158 (%)		
**Age groups (Years)**				
13-14	164 (95.3)	8 (4.7)	Reference	
15-17	175 (72.9)	65 (27.1)	7.6 (3.6 - 16.4)	<.001
18-19	46 (35.1)	85 (64.9)	37.9 (17.1 - 83.9)	<.001
**Sub-county**				
Buhanika	126 (67.4)	61 (32.6)	Reference	
Buseruka	133 (74.3)	46 (25.7)	0.7 (0.5 – 1.1)	0.146
Kitoba	126 (71.2)	51 (28.8)	0.8 (0.5 – 1.3)	0.432
**Religion**				
Anglican	97 (73.5)	35 (26.5)	Reference	
Catholic	134 (71.7)	53 (28.3)	1.1 (0.7 – 1.8)	0.719
Muslim	43 (64.2)	24 (35.8)	1.5 (0.8 – 2.9)	0.176
Pentecostal	111 (70.7)	46 (29.3)	1.1 (0.7 - 1.9)	0.600
**Education level of adolescents**				
No formal education	8 (66.7)	4 (33.3)	Reference	
Primary education	237 (74.3)	82 (25.7)	0.7 (0.2 – 2.3)	0.556
Secondary education	140 (66.0)	72 (34.0)	1.0 (0.3 – 3.5)	0.964
**Marital status of adolescents**				
Never married	343 (90.0)	38 (10.0)	Reference	
Currently Married	42 (25.9)	120 (74.1)	25.0 (15.7 – 41.6)	<.001
**Marital status of Parents**				
Currently married	239 (68.5)	110 (31.5)	Reference	
Never married	19 (95.0)	1 (5.0)	0.1 (0.0 – 0.9)	0.036
Separated	127 (73.0)	47 (27.0)	0.8 (0.5 – 1.2)	0.290
**Education level of caretaker**				
No formal education	62 (54.4)	52 (45.6)	Reference	
Primary	183 (70.9)	75 (29.1)	0.5 (0.3 – 0.8)	0.002
Secondary	140 (81.9)	31 (18.1)	0.3 (0.2 – 0.5)	<.001
**Biological parents alive**				
Yes	302 (84.1)	57 (15.9)	Reference	
No	83 (45.1)	101 (54.9)	6.4 (4.3 – 9.7)	<.001
**Parents’ economic status**				
High	110 (88.7)	14 (11.3)	Reference	
Low	275 (65.6)	144 (34.4)	4.1 (2.3 – 7.4)	<.001

### Individual factors

At a bivariate level, all factors were significantly associated with adolescent. There was a significantly high adolescent pregnancy prevalence of 74.7% among adolescents with multiple sexual partners compared to 21.1% among adolescents with no multiple partners. Those with multiple sexual partners were 9.1 times more likely to get pregnant compared to their peers with only one sexual partner COR 9.1 (95%CI 6.0-14.0). Similarly, adolescents who were not in school were 26 times more likely to get pregnant than their peers who were in school COR 26.0 (95%CI 15.9-44.1). Additionally, other individual factors that were associated with adolescent pregnancy included early marriage, control over sex, sex for material support, family planning awareness (Table 4).

**Table 4:** Unadjusted analysis analysis of factors (Individual, Family, Community and Health facility) associated with adolescent pregnancy n = 543.

Variable	Adolescent pregnancy		Crude OR (95%CI)	P-value
	No N = 385 (%)	Yes N = 158 (%)		
**Individual factors**				
**Multiple sexual partners**				
No	291 (87.9)	40 (12.1)	Reference	
Yes	94 (25.3)	118 (74.7)	9.1 (6.0 – 14.0)	<.001
**School attendance**				
In school	318 (93.0)	24 (7.0)	Reference	
Not in school	67 (33.3)	134 (66.7)	26.0 (15.9 – 44.1)	<.001
**Early marriage**				
No	341 (93.7)	23 (6.3)	Reference	
Yes	44 (14.6)	135 (85.4)	45.0 (26.5 – 78.2)	<.001
**Control over sex**				
Yes	379 (84.8)	68 (15.2)	Reference	
No	6 (6.3)	90 (93.8)	13.0 (5.2 – 19.7)	<.001
**Sex for material exchange**				
No	306 (86.7)	47 (13.3)	Reference	
Yes	79 (41.6)	111 (58.4)	9.0 (6.0 – 13.0)	<.001
**Family planning awareness**				
Yes	297 (80.5)	72(19.5)	Reference	
No	88 (50.6)	86 (49.4)	4.0 (2.7 – 6.0)	<.001
**Family planning use**				
Yes	233 (77.3)	85 (26.7)	Reference	
No	152 (67.6)	73 (32.4)	1.3 (0.9 – 1.9)	0.149
**Family related factors**				
**Discuss SRH with parents**				
Yes	273 (95.1)	14 (4.9)	Reference	
No	112 (43.8)	144 (56.3)	25.0 (13.9 – 45.3)	<.001
**Parents talk about sex education**				
Yes	267 (95.0)	14 (5.0)	Reference	
No	118 (45.0)	144 (55.0)	23.0 (12.9 – 42.0)	<.001
**Witnessed domestic violence**				
No	293 (97.0)	9 (3.0)	Reference	
Yes	92 (38.2)	149 (61.8)	52.0 (25.9 – 107.5)	<.001
**Neglected by parents/caretaker**				
No	321 (83.8)	62 (16.2)	Reference	
Yes	64 (40.0)	96 (60.0)	7.7 (5.1 – 11.8)	<.001
**Stays with parents**				
Yes	324 (90.0)	36 (10.0)	Reference	
No	61 (33.3)	122 (66.7)	18.0 (11.3 – 28.6)	<.001
**Socioeconomic status of family**				
High	110 (88.7)	14 (11.3)	Reference	
Low	275 (65.6)	144 (34.4)	4.1 (2.3 – 7.4)	<.001
**Health facility factors**				
**Provision of youth tailored health services**				
Yes	339 (76.0)	107 (24.0)	Reference	
No	46 (47.4)	51 (52.6)	3.5 (2.2 – 5.5)	<.001
**Affordability of HF services**				
Yes	355 (72.3)	136 (27.7)	Reference	
No	30 (57.7)	22 (42.3)	1.9 (1.1 – 3.4)	0.029
**Attitude of healthcare providers**				
Positive	355 (74.0)	125 (26.0)	Reference	
Negative	15 (44.1)	19 (55.9)	3.5 (1.8 – 7.3)	<.001
Neutral	15 (51.7)	14 (48.3)	2.7 (1.2 – 5.7)	0.012
**Received counselling**				
Yes	329(80.8)	78(19.2)	Reference	
No	56 (41.2)	80 (58.8)	6.0 (4.0 – 9.2)	<.001
**Felt judged by while seeking care because of age**				
No	332 (80.8)	79 (19.2)	Reference	
Yes	53 (40.2)	79 (59.8)	6.2(4.1 – 9.6)	<. 001
**Are there costs involved in accessing healthcare services**				
No	245 (77.8)	70 (22.2)	Reference	
Yes	140 (61.4)	88 (38.6)	2.2 (1.5 – 3.2)	<.001
**Community related factors**				
**Place of Residence**				
Urban	165 (78.2)	46 (21.8)	Reference	
Rural	220 (66.3)	112(33.7)	1.8 (1.2 - 2.7)	0.003
**Do media talk about issues related to adolescent pregnancy**				
No	54 (73.0)	20 (27.0)	Reference	
Yes	331 (70.6)	138 (29.4)	1.1 (0.7 – 2.0)	0.673
**Cultural influence including early marriage practices**				
No	227 (68.6)	104 (31.4)	Reference	
Yes	158 (74.5)	54 (25.5)	0.7 (0.5 – 1.1)	0.137
**Religious influence including early marriage practices**				
No	118 (68.2)	55 (31.8)	Reference	
Yes	267 (72.2)	103 (27.8)	0.8 (0.6 – 1.2)	0.345
**Peer influence including early marriage practices**				
Yes	142 (62.6)	85 (37.4)	Reference	
No	243 (76.9)	73 (23.1)	0.5 (0.3 - 0.7)	<.001

### Family-related factors

All family-related factors were significantly associated with adolescent pregnancy at a bivariate level of analysis. Adolescents neglected by parents exhibited a significantly high prevalence of adolescent pregnancies 60.0% and were 7.7 times more likely to get pregnant than their counterparts who did not experience parental neglect COR 7.7 (95%CI 5.1-11.8). Similarly, other family-related factors associated with adolescent pregnancy were; do not discuss SRH with parents, parents talk about sex education, witnessed domestic violence, do not stay with parents, and low socioeconomic status of family (Table 4).

### Health facility factors

At a bivariate level of analysis, all health facility related factors were significantly associated with adolescent pregnancy. There was a significantly high prevalence of adolescent pregnancy 59.8% among adolescents who felt judged by healthcare service providers/healthcare workers while seeking care because of their age compared to 19.2% among those who did not feel judged. Adolescents who felt judged by healthcare workers while seeking care because of their age were 6.2 times more likely to get pregnant compared to those who did not feel judged COR 6.2 (95%CI 4.1-9.6). Similarly, other adolescent pregnancy associated health facility factors included; no provision of youth tailored health services, affordability of HF services, attitude of healthcare providers, not receiving counselling, costs involved in accessing HCS (Table 4).

### Community related factors

Place of residence and peer influence including early marriage practices were the only community related factors significantly associated with adolescent pregnancy at a bivariate level of analysis. Adolescent pregnancy was more prevalent among adolescents in rural areas (33.7%) than their urban settings (21.8%) with those in rural areas being more likely to get pregnant 1.8 times higher those in urban areas COR 1.8 (95%CI 1.2-2.7). Community related factors like Religious influence including early marriage practices, Cultural influence including early marriage practices, media help mitigate issue of adolescent pregnancy, and, media talk about adolescent pregnancy were insignificantly associated with adolescent pregnancy in Hoima district (Table 4).

### Multivariable analysis

All variables with P values <0.2 at the bivariate level of analysis were selected for the multivariable analysis. The final model depicts the best fit for this analysis. All variables that remained in the multivariable model were statistically significant and they are thus significantly associated with adolescent pregnancy among girls in Hoima district.

### Factors independently associated with adolescent pregnancy

The factors that remained significantly associated with increased odds of adolescent pregnancy at multivariate analysis level included; age (18–19 years) AOR 4.4 (95%CI 1.5–12.8, p = 0.006), low parents’ economic status AOR 5.4 (95%CI 2.5–11.8, p < 0.001), multiple sexual partners AOR 8.0 (95%CI 4.5–14.2, p < 0.001), being out of school AOR 12.0 (95%CI 5.0–29.1, p < 0.001), early marriage AOR 37.0 (95%CI 13.4–107.5, p < 0.001), having no control over sex AOR 7.4 (95%CI 3.7-16.4, p < 0.001), not discussing SRH with parents AOR 8.4 (95%CI 3.3–21.5, p < 0.001), witnessed domestic violence AOR 30.0 (95%CI 12.0–77.5, p < 0.001), never received counselling AOR 5.7 (95%CI 3.6–9.0, p < 0.001), and, rural residence AOR 1.8 (95%CI 1.2–2.9, p = 0.011) (Table 5).

**Table 5:** Multivariable analysis of factors associated with adolescent pregnancy.

Variable	Crude OR (95%CI)	P-value	Adjusted OR (95%CI)	P-value
**Sociodemographic characteristics**				
**Age groups (Years)**				
13-14	Reference		Reference	
15-17	7.6 (3.6 - 16.4)	<.001	2.5 (1.0 - 6.4)	**0.045**
18-19	37.9 (17.1 - 83.9)	<.001	4.4 (1.5 - 12.8)	**0.006**
**Education level of caretaker**				
No formal education	Reference		Reference	
Primary	0.5 (0.3 – 0.8)	0.002	1.9 (1.0 - 3.7)	0.072
Secondary	0.3 (0.2 – 0.5)	<.001	0.7 (0.3 - 1.6)	0.389
**Biological parents alive**				
Yes	Reference		Reference	
No	6.4 (4.3 – 9.7)	<.001	0.8 (0.4 - 1.5)	0.513
**Parents’ economic status**				
High	Reference		Reference	
Low	4.1 (2.3 – 7.4)	<.001	5.4 (2.5 - 11.8)	**<.001**
**Individual factors**				
**Multiple sexual partners**				
No	Reference		Reference	
Yes	9.1 (6.0 – 14.0)	<.001	8.0 (4.5 - 14.2)	**<.001**
**School attendance**				
In school	Reference		Reference	
Not in school	26.0 (15.9 – 44.1)	<.001	12.0 (5.0 - 29.1)	**<.001**
**Early marriage**				
No	Reference		Reference	
Yes	45.0 (26.5 – 78.2)	<.001	37.0 (13.4 - 107.5)	**<.001**
**Control over sex**				
Yes	Reference		Reference	
No	13.0 (5.2 – 19.7)	<.001	7.4 (3.7 - 16.4)	**<.001**
**Sex for material exchange**				
No	Reference		Reference	
Yes	9.0 (6.0 – 13.0)	<.001	1.3 (0.6 - 2.8)	0.542
**Family planning awareness**				
Yes	Reference		Reference	
No	4.0 (2.7 – 6.0)	<.001	5.5 (2.4 - 12.8)	**<.001**
**Family planning use**				
Yes	Reference		Reference	
No	1.3 (0.9 – 1.9)	0.149	1.2 (0.8 – 1.9)	0.324
**Family-related factors**				
**Discuss SRH with parents**				
Yes	Reference		Reference	
No	25.0 (13.9 – 45.3)	<.001	8.4 (3.3 - 21.5)	**<.001**
**Parents talk about sex education**				
Yes	Reference		Reference	
No	23.0 (12.9 – 42.0)	<.001	5.8 (2.7 - 12.3)	**<.001**
**Witnessed domestic violence**				
No	Reference		Reference	
Yes	52.0 (25.9 – 107.5)	<.001	30.0 (12.0 - 77.5)	**<.001**
**Neglected by parents/caretaker**				
No	Reference		Reference	
Yes	7.7 (5.1 – 11.8)	<.001	0.7 (0.4 - 1.4)	0.338
**Stays with parents**				
Yes	Reference		Reference	
No	18.0 (11.3 – 28.6)	<.001	1.1 (0.5 - 2.7)	0.844
**Health facility related factors**				
**Provision of youth tailored health services**				
Yes	Reference		Reference	
No	3.5 (2.2 – 5.5)	<.001	4.3 (2.2 - 8.7)	**<.001**
**Affordability of HF services**				
Yes	Reference		Reference	
No	1.9 (1.1 – 3.4)	0.029	0.7 (0.3 - 1.7)	0.450
**Attitude of healthcare providers**				
Positive	Reference		Reference	
Negative	3.5 (1.8 – 7.3)	<.001	1.1 (0.5 - 2.8)	0.795
Neutral	2.7 (1.2 – 5.7)	0.012	0.7 (0.2 - 1.7)	0.390
**Received counselling**				
Yes	Reference		Reference	
No	6.0 (4.0 – 9.2)	<.001	5.7 (3.6 - 9.0)	**<.001**
**Felt judged by while seeking care because of age**				
No	Reference		Reference	
Yes	6.2 (4.1 – 9.6)	<. 001	4.9 (2.9 - 8.3)	**<.001**
**Are there costs involved in accessing healthcare services**				
No	Reference		Reference	
Yes	2.2 (1.5 – 3.2)	<.001	1.9 (1.2 - 3.0)	**0.005**
**Community related factors**				
**Place of Residence**				
Urban	Reference		Reference	
Rural	1.8 (1.2 - 2.7)	0.003	1.8 (1.2 - 2.9)	**0.011**
**Peer influence including early marriage practices**				
Yes	Reference		Reference	
No	0.5 (0.3 – 0.7)	<.001	0.7 (0.5 - 1.1)	0.156

## Discussion

This study determined the prevalence of adolescent pregnancies and described the socio-demographic, individual, family, community and Health facility factors associated with pregnancy among adolescent girls aged 13–19 years in Hoima district, Uganda. The findings indicate adolescent pregnancy remains an important public health challenge in Hoima district. The prevalence observed suggests persistent geographical inequalities despite national efforts to reduce adolescent pregnancy.

The higher likelihood of pregnancy among older adolescents may reflect cumulative exposure to sexual relationships, greater social pressure to enter marriage, and increasing expectations of childbearing as girls approach adulthood. The finding is consistent with studies from Uganda and Ethiopia, which demonstrate that pregnancy risk increases with age due to prolonged exposure to sexual activity and higher likelihood of marriage [6,8]. Unlike younger adolescents, older girls in rural Uganda are more likely to be perceived as socially “ready” for marriage and childbearing, even when still legally minors. This socio-cultural expectation may explain why age remains a strong predictor despite increased awareness of sexual and reproductive health information among older adolescents.

Poverty has been consistently associated with adolescent vulnerability in many settings. Girls from economically disadvantaged households may engage in early sexual relationships as a coping strategy for unmet basic needs, school fees, or material support as seen in a related Ugandan socioeconomic vulnerability models [9]. Similar associations have been reported in Uganda, Tanzania, and other low-resource settings, where poverty exacerbates school dropout, early marriage, and transactional sex [7,10,11]. These findings reinforce the need to address structural economic factors alongside behavioral interventions.

Evidence from Ethiopia and Sri Lanka suggest that being in school not only delays marriage and sexual debut but also provides structured supervision, access to reproductive health information, and future aspirations that discourage early childbearing [8,11,12]. In Hoima district, school dropout is often associated with poverty, early marriage, and domestic responsibilities, creating a cyclical relationship in which leaving school and adolescent pregnancy frequently coexist and reinforce social disadvantage. This interaction may explain why school attendance remains one of the most consistent predictors across diverse settings [13].

Similar associations between early marriage and adolescent pregnancy have been reported across Uganda and other sub-Saharan African countries (SSA), where marriage is often accompanied by expectations of immediate childbearing [13,14]. Married adolescents may have reduced autonomy over reproductive decisions and less opportunity to delay pregnancy through contraceptive use. Although Uganda has legal frameworks prohibiting child marriage, implementation remains challenging in some rural communities. In Hoima District, prevailing social norms that link marriage with motherhood may help explain the strong association observed between early marriage and adolescent pregnancy

Similar findings have been reported in Ethiopia and other SSA countries. These findings suggest heightened exposure to unprotected sex, coercion, and inconsistent contraceptive use [11,15,16]. Limited sexual autonomy among adolescent girls may result from gender power imbalances, economic dependency, or partner manipulation, all of which reduce the ability to negotiate safe sex and delay pregnancy [11]. In rural Hoima, girls may lack bargaining power within relationships, reducing their ability to negotiate condom use or delay sex, thereby increasing pregnancy risk even when awareness of contraception exists.

Findings from Zambia and Kenya suggest that open family communication is associated with healthier sexual behaviours and delayed sexual initiation among adolescents [17,18]. Open parent–child communication may provide adolescents with accurate information and guidance that may support informed reproductive health decisions. In rural settings such as Hoima district, cultural discomfort around discussing sexuality may limit these conversations, leaving adolescents more dependent on peers or other informal sources of information. This challenge may be particularly important where access to adolescent-friendly health services is limited

Evidence from Brazil and Uganda has linked household violence to adverse adolescent reproductive outcomes [18,19]. Witnessing violence within the household may normalize harmful gender norms, undermine emotional well-being, and increase susceptibility to risky sexual behavior. Emotional distress, low self-esteem, and disrupted family support systems may drive adolescents toward early sexual relationships as coping mechanisms. In Hoima District, domestic violence often coexists with alcohol misuse and economic stress, potentially compounding its impact on adolescent girls’ vulnerability.

Similar findings have been reported in Nigeria, where limited access to SRH counselling has been associated with higher levels of unprotected sexual activity and unintended pregnancy among adolescents [20]. Counselling may provide adolescents with accurate information on contraception, fertility awareness, and negotiation skills, enabling informed decision-making. In Hoima District, limited counselling opportunities may reflect shortages of trained personnel, high patient loads, and inadequate prioritization of adolescent-specific services. Without counselling, adolescent girls may rely on peers or informal sources for information, increasing susceptibility to misinformation and risky sexual behavior.

Evidence from sub-Saharan Africa suggest that affordability remains an important consideration in adolescents’ utilization of sexual and reproductive health services [21]. Even when public health services are nominally free, indirect costs such as transportation, registration fees, and opportunity costs related to time away from school or household responsibilities can deter adolescents from seeking care. A study in Nigeria has similarly shown that adolescents from low-income households are less likely to access contraceptive services due to cost-related barriers [20]. In Hoima district, where poverty levels remain high and health facilities are sparsely distributed, these costs may disproportionately affect rural adolescents, limiting timely access to preventive SRH services, which may partly explain the observed association.

Perceived judgement or negative attitudes from healthcare providers have been identified as important barrier to effective SRH service utilization among adolescents. This finding aligns with studies from Uganda which document how stigma, breaches of confidentiality, and moralizing attitudes discourage adolescents from accessing SRH services [7,14]. In conservative rural settings like Hoima, fear of being reprimanded or reported to parents can further intensify adolescents’ reluctance to seek care. Such experiences undermine trust in the health system and highlight the need for provider training in adolescent-friendly, non-judgmental care.

Similar findings have been reported in Zambia [17]. Rural residence often reflects broader structural disadvantages, including limited access to quality education, adolescent-friendly health services, and economic opportunities. In Hoima district, rural communities are also characterized by strong cultural norms that support early marriage and childbearing, which may override formal policy restrictions. Although some multi-country analyses have reported lower adolescent pregnancy risk in rural settings due to reduced exposure to sexualized media, the present findings suggest that in Hoima, the effects of poverty, service inaccessibility, and entrenched social norms may outweigh any potential protective factors [2].

## Study limitations

The cross-sectional study design makes it impossible to determine temporal relationships between exposure and adolescent pregnancy because causality cannot be established using this study design. Self-reporting for the pregnant status of the respondents as well as other sensitive data, which could be subject to recall and social desirability biases. These biases were minimized by conducting an interview under a trained interviewer in a private setting using memory aids. Although multicollinearity assessment indicated no evidence of problematic multicollinearity, residual confounding from unmeasured factors cannot be entirely ruled out. In addition, some estimates were associated with relatively wide confidence intervals, which may reflect sparse data within some exposure categories, potentially leading to unstable estimates. Consequently, the magnitude of these associations should be interpreted with caution, although the direction of the observed relationships remains informative. Lastly, the study used a probability sample design through multistage probability sampling; however, household listing and community registers might have excluded mobile adolescents. These limitations should be considered when interpreting the findings

## Conclusion and recommendations

Adolescent pregnancy remains a major public health concern in Hoima district, with a prevalence of 29.1%. Several factors were independently associated with adolescent pregnancy. These included older age of adolescents (18–19 years), lower parental socio-economic status, having multiple sexual relationships, being out of schools, early marriage, lack of control over sexual decision-making, inadequate communication between parents and their daughters about sexual matters, witnessing domestic violence in the home, not receiving counselling services, and living in rural areas.

These findings suggest that adolescent pregnancy is associated with a combination of individual, family, community, and health system factors. Therefore, policymakers and stakeholders should implement multisectoral interventions that aim at ensuring that girls remain in schools, avoid getting married at an early age, have access to adolescent-friendly health care providers without any stigma, foster open communication within families, and address poverty in households. The findings should be incorporated into various policies to address the problem of adolescent pregnancies in the district.

## Supporting information

S1 File Dataset used for analysis. An anonymized dataset containing variables used in the quantitative analysis of the prevalence and associated factors of adolescent pregnancy in Hoima.
(XLSX)

## Abbreviations

AOR: Adjusted Odds Ratio
COR: Crude Odds Ratio
DHS: Demographic Health Survey
IQR/SD: Interquartile Range/Standard Deviation
PRB: Population Reference Bureau
SRH: Sexual and Reproductive Health
SSA: Sub-Saharan Africa
UBOS: Uganda Bureau of Statistics
UNICEF: United Nations International Children’s Emergency Fund
WHO: World Health Organization.
